# Advancing the diagnosis of cardiac electrophysiological disorders in diabetes: integrating clinical, imaging, and molecular insights

**DOI:** 10.3389/fmed.2026.1777638

**Published:** 2026-02-03

**Authors:** Venkata Nagaraj Kakaraparthi, Paul Silvian Samuel, Lalitha Kakaraparthi, Vamsi Krishna Gannamaneni, Kumar Gular

**Affiliations:** 1 Department of Medical Rehabilitation Sciences, College of Applied Medical Sciences, King Khalid University, Abha, Saudi Arabia; 2 Department of Physiotherapy, University of Hail, Hail, Saudi Arabia

**Keywords:** AI-enabled arrhythmia detection, diabetic cardiac electrophysiopathy, electrophysiological risk stratification, multimodal cardiac diagnostics, precision cardiometabolic medicine

## Abstract

Diabetes mellitus is a systemic metabolic disorder associated with an increased risk of cardiac electrophysiological abnormalities, including atrial and ventricular arrhythmias, conduction disturbances, and autonomic dysfunction. These complications contribute substantially to morbidity and mortality but are frequently underrecognized due to limitations of conventional diagnostic approaches that rely primarily on surface electrocardiography and intermittent monitoring. Growing evidence suggests that electrophysiological instability in diabetes arises from a complex interaction of metabolic dysregulation, microvascular impairment, inflammation, and molecular alterations that are not fully captured by traditional electrical assessments alone. Recent advances in cardiovascular imaging, molecular diagnostics, and artificial intelligence–driven analytics provide new opportunities to enhance the detection, risk stratification, and characterization of diabetic cardiac electrophysiological disorders. This Perspective discusses the evolving clinical spectrum of electrophysiological abnormalities in diabetes, highlights the shortcomings of existing diagnostic paradigms, and explores emerging innovations that integrate clinical assessment with advanced imaging and molecular insights. We propose an integrative diagnostic framework that leverages multimodal data and digital health technologies to enable earlier identification of high-risk individuals and support precision cardiology approaches. Advancing such integrated diagnostic strategies may improve clinical decision-making, facilitate personalized management, and ultimately reduce the burden of cardiac electrophysiological complications in people with diabetes.

## Introduction

Diabetes mellitus is a systemic metabolic disorder with well-recognized vascular and myocardial consequences. Beyond structural heart disease, individuals with diabetes are at increased risk of cardiac electrophysiological abnormalities, including atrial and ventricular arrhythmias, conduction disturbances (1), and autonomic dysfunction (2). These abnormalities contribute substantially to morbidity, sudden cardiac death, and reduced quality of life. However, electrophysiological complications in diabetes are frequently underdiagnosed or detected late, largely due to the limitations of conventional diagnostic approaches (3).

Emerging evidence suggests that electrical instability in the diabetic heart arises from a complex interplay of metabolic dysregulation, microvascular dysfunction, inflammation, and subtle myocardial remodelling (4). Advances in imaging, molecular diagnostics, and data-driven technologies now offer opportunities to move beyond isolated electrical measurements toward integrated diagnostic frameworks. This perspective highlights the need to advance diagnostic strategies for diabetic cardiac electrophysiological disorders by combining clinical assessment with innovative imaging and molecular insights.

In this Perspective, we propose that advancing the diagnosis of diabetic cardiac electrophysiological disorders requires a paradigm shift from isolated electrical assessments toward integrative, multimodal diagnostic strategies. In our view, combining clinical electrophysiology with advanced imaging, molecular profiling, and data-driven analytics offers a more comprehensive understanding of arrhythmogenic risk in diabetes.

### Clinical spectrum of cardiac electrophysiological disorders in diabetes

Patients with diabetes exhibit a broad range of electrophysiological abnormalities (5). Atrial fibrillation is more prevalent in diabetic populations and is associated with poorer outcomes and increased stroke risk. Ventricular arrhythmias, prolonged QT intervals, and conduction delays have also been reported, particularly in individuals with long-standing disease or poor glycaemic control (6).

Autonomic neuropathy represents another important contributor, leading to heart rate variability abnormalities and increased susceptibility to malignant arrhythmias. Despite these risks, many electrophysiological disturbances remain clinically silent until advanced stages. Standard diagnostic tools such as resting electrocardiography and intermittent monitoring may fail to capture transient or early abnormalities, underscoring the need for more sensitive and integrative diagnostic approaches (7).

### Limitations of conventional diagnostic approaches

Conventional electrophysiological diagnostics primarily rely on surface ECGs, Holter monitoring, and invasive electrophysiology studies. While valuable, these tools provide limited insight into the underlying myocardial substrate contributing to electrical instability. They often capture electrical manifestations without addressing the metabolic, microstructural, or molecular drivers of disease (8).

Additionally, risk stratification based solely on electrical parameters may overlook patients at high risk of arrhythmia development. This diagnostic gap highlights the need to complement electrical assessment with modalities capable of characterizing myocardial health, biological signalling pathways, and early disease processes in diabetic hearts.

### Emerging imaging and molecular diagnostic innovations

Recent advances in cardiovascular imaging have enhanced the ability to assess myocardial structure and function beyond gross anatomy. Techniques such as cardiac magnetic resonance imaging, advanced echocardiographic strain analysis, and myocardial tissue characterization can detect subtle changes associated with diabetic cardiomyopathy and electrical vulnerability (9).

Parallel developments in molecular diagnostics have identified circulating biomarkers related to inflammation, fibrosis, oxidative stress, and ion-channel regulation that may reflect arrhythmogenic risk (10). While not replacing electrophysiological testing, these biomarkers offer complementary insights into disease mechanisms (11).

Artificial intelligence and machine learning approaches further enable the integration of complex datasets, including imaging outputs, molecular profiles, and electrophysiological signals. AI-driven analysis has shown promise in detecting subclinical abnormalities, enhancing pattern recognition, and improving predictive accuracy for adverse cardiac events (12).

### Toward an integrative diagnostic framework

A shift toward integrative diagnostics is essential for advancing the detection and management of electrophysiological disorders in diabetes. Combining clinical assessment, electrophysiological testing, imaging findings, and molecular indicators allows for a more comprehensive understanding of disease processes. Such frameworks can support earlier identification of high-risk individuals, improve phenotyping, and guide personalized management strategies (13).

We suggest that the diagnostic modalities outlined should not function as parallel tools but rather within a coordinated, data-integrative structure. In our view, aligning electrophysiological signals with imaging-based myocardial characterization, molecular biomarkers, and AI-enabled analytics provides incremental value by enabling earlier risk stratification and more precise clinical decision-making in patients with diabetes.

Beyond diagnostic integration, we further propose that this framework enables a dynamic, learning-oriented diagnostic ecosystem rather than a static assessment model. By continuously integrating longitudinal electrophysiological signals, imaging-derived myocardial features, and evolving molecular profiles, the framework can support adaptive risk stratification over time. In our view, such an approach shifts diagnostic practice from episodic detection to anticipatory monitoring, allowing clinicians to identify trajectory-based risk patterns and intervene earlier in the course of diabetic cardiac electrophysiological disease.

**Figure 1:**
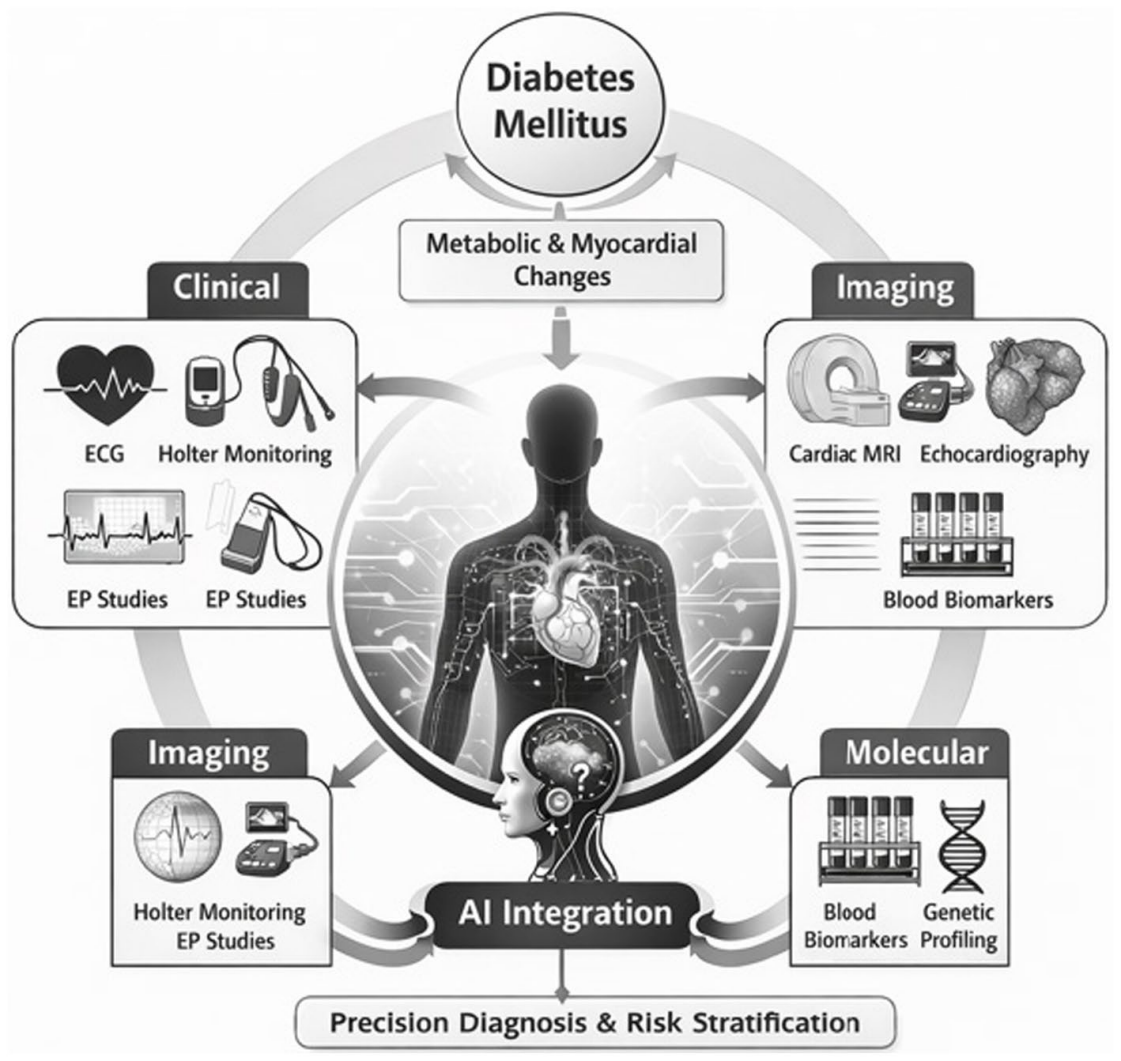
Illustrates an integrative diagnostic framework linking clinical electrophysiological assessment with advanced imaging, molecular insights, and data-driven technologies to improve detection and risk stratification of cardiac electrophysiological disorders in diabetes.

Rather than focusing on isolated diagnostic modalities, future approaches should emphasize multimodal data integration supported by digital health platforms and AI-enabled decision tools. This paradigm aligns with precision medicine principles and addresses the heterogeneity inherent in diabetic cardiac complications. (Figure 1).

Unlike routine diagnostic practice that relies largely on isolated electrocardiographic findings or episodic rhythm monitoring, the proposed framework integrates electrophysiological data with advanced imaging, molecular biomarkers, and AI-enabled analytics. In our view, this coordinated approach enables a more comprehensive assessment of arrhythmogenic risk in diabetes. The framework promotes interdisciplinary collaboration, supporting earlier risk identification and more personalized diagnostic pathways.

### Future directions and innovation opportunities

Future research should prioritize longitudinal studies integrating electrophysiological data with imaging and molecular markers to establish predictive models of arrhythmic risk in diabetes. Validation of AI-assisted diagnostic tools in real-world clinical settings is also critical. Additionally, collaborative efforts between clinicians, pathologists, engineers, and data scientists will be essential to translate technological innovation into clinical practice.

Importantly, diagnostic innovation should remain patient-centered, cost-effective, and scalable, particularly in regions with high diabetes prevalence. Integrative diagnostic strategies have the potential not only to improve detection but also to inform targeted prevention and therapeutic interventions.

From a translational perspective, this framework may assist clinicians in moving from reactive arrhythmia detection toward proactive, risk-informed diagnostic planning in patients with diabetes.

## Conclusion

Cardiac electrophysiological disorders represent a significant yet underrecognized complication of diabetes mellitus. Advances in imaging, molecular diagnostics, and artificial intelligence provide an opportunity to move beyond conventional electrical assessments toward integrated diagnostic frameworks. By combining clinical, imaging, and molecular insights, future diagnostic strategies can enhance early detection, improve risk stratification, and support precision cardiology in diabetic populations.

We suggest that adopting integrative diagnostic frameworks represents a necessary step toward precision cardiometabolic medicine. In our view, such approaches have the potential to transform current diagnostic paradigms by enabling earlier identification of high-risk individuals and supporting personalized management strategies.

## Data Availability

The original contributions presented in the study are included in the article/supplementary material, further inquiries can be directed to the corresponding author.
